# Bibliometric analysis of Oropouche research: impact on the surveillance of emerging arboviruses in Latin America

**DOI:** 10.12688/f1000research.10936.2

**Published:** 2017-03-17

**Authors:** Carlos Culquichicón, Jaime A. Cardona-Ospina, Andrés M. Patiño-Barbosa, Alfonso J. Rodriguez-Morales

**Affiliations:** 1 Emerge, Emerging Diseases and Climate Change Research Unit, Universidad Peruana Cayetano Heredia, Lima, Peru; 2 Scientia Clinical and Epidemiological Research Institute, Trujillo, Peru; 3 Public Health and Infection Research Group, Faculty of Health Sciences, Universidad Tecnologica de Pereira, Pereira, Risaralda, Colombia; 4 Colombian Collaborative Network of Zika and other Arboviruses (RECOLZIKA), Pereira, Risaralda, Colombia

**Keywords:** Oropouche, arbovirus, epidemiology, public health, travelers, Latin America

## Abstract

Given the emergence and reemergence of viral diseases, particularly in Latin America, we would like to provide an analysis of the patterns of research and publication on Oropouche virus (OROV). We also discuss the implications of recent epidemics in certain areas of South America, and how more clinical and epidemiological information regarding OROV is urgently needed.

## Introduction

The Oropouche virus (OROV) is an emerging arbovirus that threatens the Amazon region of Brazil, Peru, and Venezuela ^1^. The coexistence of this pathogen with other long-term circulating arboviruses, such as dengue virus (DENV), West Nile virus (WNV), Venezuelan Equine Encephalitis (VEEV) and yellow fever virus (YFV), as well as emerging arboviruses such as chikungunya (CHIKV), Zika (ZIKV) and Mayaro virus (MAYV), may hinder clinical diagnosis and successful vector-control strategies ^2^. Research is essential to be able to manage this complex scenario. As has been highlighted by Ballabeni and Boggio ^3^, bibliometric analyses of publications on emerging and reemerging viral diseases are important as they may lead to insights on how the global scientific and health communities react to outbreaks. We aimed to conduct a bibliometric analysis of OROV research and the impact on the surveillance of emerging and re-emerging arboviruses in Latin America.

## Methods

A bibliometric study was done about OROV scientific production, with a focus on Latin America. We searched in three important regional and international databases (all of them in English): Science Citation Index Expanded (SCI-E), Scopus, Medline (via GoPubMed®), LILACS, SciELO and IMBIOMED.

This search strategy used the following key words (MeSH, Medical Subject Headings): “Oropouche” AND “Latin America”, “Oropouche” AND “Argentina”, “Oropouche” AND “Colombia”, and the same way with the rest of the Latin American countries. Also, “OROV” was used instead of Oropouche for additional searches. All study types were included (original articles, reviews, case reports, editorials) and were categorized by year, international cooperation, city and institution, journal and authors with major contribution. Searches were done from May 30 to June 30, 2015.

Data was tabulated and analyzed in Excel 2007® for Windows 7® ( Dataset 1 ^4^), summarizing quantitative variables with means and interquartile ranges (IQRs), and qualitative variables with proportions.

Raw data obtained from bibliographical databases (Medline, Scopus and SCI-E) Click here for additional data file. Copyright: © 2017 Culquichicón C et al. 2017 Data associated with the article are available under the terms of the Creative Commons Zero "No rights reserved" data waiver (CC0 1.0 Public domain dedication).

## Results

**Figure 1.**
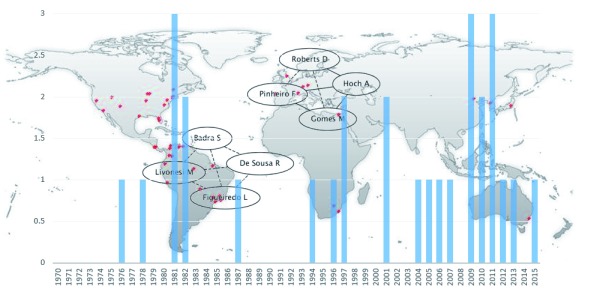
Graphical representation of major international research networks involved in research on Oropouche (via GoPubMed®) and research trends from 1970 to 2015.

A total of 260 related records were retrieved in our search; from these, 97 manuscripts were recovered in Scopus (55% from Brazil, 28% from US, and 11% from Peru); 83 articles were recovered from Medline (43% from Brazil, 18% from US, and 6% from Peru) and 80 articles were recovered from SCI-E (61% from Brazil, 35% from US, and 15% from Peru) ( Table 1). As observed in Medline, publications on OROV never reached more than 3 articles per year ( Figure 1). Analyzing this database, it can be observed that Brazil has the more productive and cooperative research groups in Latin America ( Figure 1).

**Table 1.** Countries with highest scientific output on Oropouche, using data taken from Science Citation Index-Expanded, Scopus and/or Medline (up to June 30, 2015).

Rank	Country	Number of articles	Database with highest number of articles
1	Brazil	53	SCOPUS
2	United States	28	SCI-E
3	Peru	12	SCI-E
4	Trinidad and Tobago	8	SCOPUS
4	United Kingdom	8	SCI-E
5	Canada	7	SCOPUS
6	Germany	5	SCI-E
7	Argentina	3	SCI-E
7	Paraguay	3	SCI-E
8	Argentina	2	SCOPUS
8	Netherlands	2	SCI-E
8	Ecuador	2	SCI-E
9	Australia	1	SCOPUS
9	Bolivia	1	SCOPUS
9	Australia	1	SCI-E
9	Argentina	1	Medline
9	Czech Republic	1	Medline
9	Norway	1	Medline
9	Trinidad and Tobago	1	Medline

**Figure 2.**
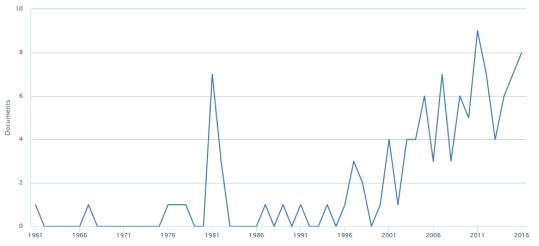
Research trends on Oropouche virus from 1960 to 2016. Data taken from Scopus.

**Figure 3.**
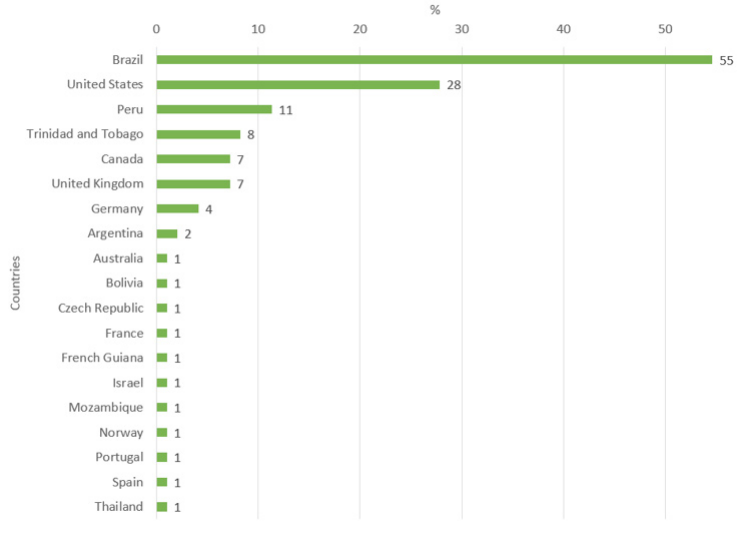
Research on Oropouche virus, ranked by contributing countries (%). Data taken from Scopus.

**Figure 4.**
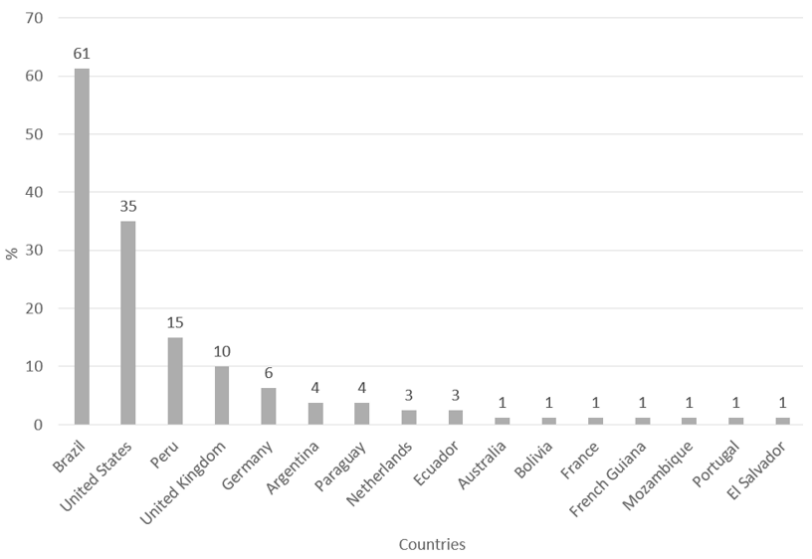
Research on Oropouche virus, ranked by contributing countries (%). Data taken from SCI-E.

For Scopus, the annual average number of articles published up to 2014 was 5 (IQR: 1–17) ( Figure 2). In June 2015, only two articles had been published that year. Nevertheless, after 1996, although not uniform, there was an increasing trend in the number of articles published on OROV per year, reaching 9 in 2011 ( Figure 2). At Scopus 19 countries contributed to the publication of at least 1 paper during the study period ( Figure 3). For SCI-E, the annual average number of articles published up to 2014 was 6.2 (IQR: 1–20), with 16 countries contributing to the publication of at least 1 paper during the study period ( Figure 4). 

“Universidade de Sao Paulo” in Sao Paulo, Brazil, was the institution with the most prolific research contribution, and “Figueiredo, L.T.M” was the author with the longest record in Oropouche research, with 12 articles ( Figure 1 and Figure 2). The greatest H-indexes for Oropouche issues came from Brazil (H-index=12, 431 citations), the United States of America (H-index=10, 339 citations), Peru (H-index=9, 234 citations), United Kingdom (H-index=6, 144 citations), Canada (H-index=5, 155 citations) and Trinidad and Tobago (H-index=4, 92 citations).

In the case of LILACS, only 35 articles were retrieved, 26 of them from Brazil, 3 from Peru, 1 from Argentina, 1 from Paraguay and 1 from Venezuela. Of them, 10 were published in the journal Revista da Sociedade Brasileira de Medicina Tropical. The year with the highest number of articles published at this database was 1982 with 5.

At SciELO, 22 articles were located, 18 from the Brazil collection and 1 from Peru collection, published mainly between 2015–2016 (6 articles), being 12 in English, 8 in Portuguese and 2 in Spanish.

Finally, at IMBIOMED none results were found.

## Discussion

OROV outbreaks increase when the rainy season starts (January to June) in endemic areas, where the population density of *Culicoides paraensis* is high ^1^. In fact, the OROV dispersion routes and its genetic diversity ^5^ impacted on the growth of scientific publications, as well as on the international collaboration on this topic. On the 2 ^nd^ of May 2016, the Ministry of Health of Peru reported 57 cases of OROV fever ^6^. Most cases originate in towns located in the northern part of the Cusco Region, which is situated in the Amazon rainforest. 79% were detected in January, with only 7% and 14% of the cases being identified in February and March, respectively. There were no fatalities and all patients have recovered following symptomatic treatment. In February 2016, a field mission to the Madre de Dios Region conducted jointly by the Ministry of Health of Peru and PAHO/WHO revealed a mixed outbreak of dengue (DENV-2) and OROV. While Madre de Dios already experienced an outbreak of OROV fever in 1994, at the time of the mission in February, this latest outbreak was of a higher magnitude, with 120 confirmed cases ^6^. Cases have also been reported in other nearby countries such as Panama, Trinidad and Tobago and Brazil, and very recently in Venezuela (2016) ^1, 7^. It highlights the potential for expansion of OROV and other related reassortant viruses to other countries in the region, such as Colombia, Venezuela and Ecuador, amongst others in South and Central America.

Despite this epidemiological situation, research on OROV is far below the level of research on other emerging arboviruses in Latin America such as CHIKV (6,344 articles recovered) or ZIKV ^8, 9^. This lack of published studies does not allow evidence-based decision-making on public health policies. More clinical and epidemiological information regarding OROV is urgently needed. Especially in highly vulnerable areas, such as those where other arboviruses (CHIKV, ZIKV, DENV) are circulating because vector and climate conditions are suitable for transmission ^10– 13^, research on OROV deserves more incentives among institutions, so that specific laboratory tests can be designed and more knowledge on this this emerging arbovirus can be gathered properly ^2, 10– 13^. Currently, differential diagnosis of these arboviruses (CHIKV, DENV, ZIKV, MAYV) poses a significant challenge ^10^, especially in the scenario of co-circulation and/or syndemics with emerging and circulating arboviruses, or even in the scenario of co-infections ^10– 13^.

The current bibliometric analysis was not restricted to Latin American countries. Although this is an arbovirus that emerged in the region, there is interest from international groups to cooperate in the research on OROV from outside Latin America. Even more, Latin America can be the source of imported cases in North America and Europe, as has been happening not just with arboviral diseases ^9, 10^, but also with Chagas disease. When revising the bibliometric analysis to take into account such a situation, it can be seen that there are research groups outside Latin America contributing to research about this disease. Then, countries such as USA, Canada andSpain, among others, would be concerned about the potential impact of the spread of this arbovirus outside Latin America ^14^.

In conclusion, Brazil is leading the initiative on OROV research. Probably this country is the main contributor since its budget for science is one of the highest in Latin America, and not necessarily because it suffers in a greater proportion of this problem. Nevertheless, OROV infection is a differential diagnosis in the Amazonas area of Brazil. Besides this, international research networks should be expanded to gain a full understanding of this arboviral disease and explore its potential expansion and impact. To do this, the epidemic dispersion, transmission cycle, molecular epidemiology, pathogenesis, and clinical features of OROV need to be studied.

## Data availability

The data referenced by this article are under copyright with the following copyright statement: Copyright: © 2017 Culquichicón C et al.

Data associated with the article are available under the terms of the Creative Commons Zero "No rights reserved" data waiver (CC0 1.0 Public domain dedication). 

**Dataset 1: Raw data obtained from bibliographical databases (Medline, Scopus and SCI-E).**

**DOI,** 10.5256/f1000research.10936.d152949 ^4^
