# Supplementary material for: Bibliometric analysis of Oropouche research: impact on the surveillance of emerging arboviruses in Latin America
Source: F1000Res. 2017 Mar 17;6:194. Originally published 2017 Feb 28. [Version 2] doi: 10.12688/f1000research.10936.2 (PMC5357042; doi:10.12688/f1000research.10936.2)
Supplement: Peer review history

# Peer review: Referee response for version 2

**DOI:** 10.5256/f1000research.12074.r20659

**Published:** 2017-04-10

**Authors:** Rodrigo Nogueira Angerami

**Affiliations:** 1 Faculty of Medical Sciences, Department of Clinical Medicine, Clinical Hospital, University of Campinas (UNICAMP), Campinas, Brazil

The present manuscript discusses an important issue in the current epidemiological scenario: the importance of arboviral diseases as a health problem worldwide. Despite the important of the recognition and knowledge of certain diseases in this group, they present quite a heterogeneous public health challenge to sanitary authorities, both as a medical problem for healthcare professions, and a risk to the health of the general populace. While dengue and, more recently, Zika and chikungunya, reached a high level of recognition, priority, "notoriety", Oropouche fever, certainly, does not appears to be part of this group.

 The subject of this paper is an interesting insight regarding how much Oropouche is also potentially put down as an object of study and interest of research. Additionally, it identifies where the hotspots of scientific production are, who are potential partners for scientific cooperation projects and what the main databases for bibliographic revision are.

 Some questions, however, must be addressed:

 - In the introduction, authors included West Nile, besides dengue (as an example) as a long-term circulating virus. Not in Latin America, especially if considering the South America.

 - Why not include other indicators as to the patterns of study design (review, case series, case report ...) and the journals impact factor.

 - It would be interesting to correlate/compare, by country and year/period, the number of described/reported cases in the papers with those reported/notified (or not) found in the official databases.

 - In table 1, it would be recommended to include a "Total" in the last line and a "%" column besides "number of articles" column.

 - It is potentially confusing to have "geographical representation of major international research networks" and "research trends" in the same figure (figure 1).

 - As a suggestion, in the discussion there is a lot of text on a single example, at Peru. It would be more interesting and illustrative to describe the occurrence along the time of first cases and, specifically, outbreaks in Latin American countries.

I have read this submission. I believe that I have an appropriate level of expertise to confirm that it is of an acceptable scientific standard.

# Peer review: Referee response for version 2

**DOI:** 10.5256/f1000research.12074.r21111

**Published:** 2017-03-20

**Authors:** Luis Cuauhtémoc Haro-García

**Affiliations:** 1 Department of Public Health, Faculty of Medicine, National Autonomous University of Mexico (UNAM), Mexico City, Mexico

I have no further comments.

I have read this submission. I believe that I have an appropriate level of expertise to confirm that it is of an acceptable scientific standard.

# Peer review: Referee response for version 1

**DOI:** 10.5256/f1000research.11788.r20599

**Published:** 2017-03-15

**Authors:** Luis Cuauhtémoc Haro-García

**Affiliations:** 1 Department of Public Health, Faculty of Medicine, National Autonomous University of Mexico (UNAM), Mexico City, Mexico

The title of the manuscript seems to me appropriate, the summary is clear enough as to the purpose of the study. The design is adequate and sufficiently explained. The discussion chapter is balanced in such a way that it will be able to draw the attention of the scientific community on this emerging arbovirus in the region of Latin America.

 Further Comments:

 - If some of the contributions for non-Latin American countries (the United States, Canada, Australia, Norway, Thailand and others), were carried out in a Latin America country or in their own country, please say the reason for including them in the bibliometric analysis.

 - Although the bibliometric analysis that was performed apparently fulfills the objective of the study ----but it includes results of non-Latin American countries---it would have been interesting to know if the scientific production of nations of other regions of the world share the concern they should be taking into account more the emergency and re-emergence of this arbovirus.

 - It would be appropriate to discuss, by the authors, that Brazil probably is the main contributor to the issue because its budget for science is one of the highest in Latin America, and not because it suffers in a greater proportion of this problem, or if there are other reasons.

 - It would have been desirable to consider some regional bibliographic databases such as LILACS and IMBIOMED, and not just English-speaking ones for a best bibliometric analysis, or to indicate why they were not taken into account.

 I have read this submission and believe that I have the level of expertise to indicate that the study has the sufficient scientific standard

I have read this submission. I believe that I have an appropriate level of expertise to confirm that it is of an acceptable scientific standard.

# Response

**Published:** 2017-03-15

**Authors:** Alfonso Rodriguez-Morales

**Affiliations:** Universidad Tecnológica de Pereira, Colombia

Dear Dr. Haro-García

 Thanks for your comments.

 The current bibliometric analysis was not restricted to Latin American countries, although this is an arbovirus that emerged in the region, there is interest from international groups to cooperate in the research on OROV from outside Latin America. Even more, Latin America can be the source of imported cases in North America and Europe, as has been happening not just with arboviral diseases, but specially with Chagas disease. When revising in bibliometric analysis such situation, also is see that there research groups outside Latin America contributing with this disease (Delgado-Osorio N, Vera-Polanía F, López-Isaza AF, Martínez-Pulgarin DF, Murillo-Abadia J, Muñoz-Urbano M, Cardona-Ospina JA, Bello R, Lagos-Grisales GJ, Villegas S, Rodríguez-Morales AJ. Bibliometric assessment of the contributions of literature on Chagas disease in Latin America and the Caribbean. Recent Pat Antiinfect Drug Discov 2014 Sep-Dec; 9(3):202-208). Then, countries such as USA, Canada, Spain, among others would be concerned about the potential impact of the spread of this arbovirus outside Latin America.

 Certainly, we agree that Brazil probably is the main contributor to the issue because its budget for science is one of the highest in Latin America, and not necessarily because it suffers in a greater proportion of this problem. Nevertheless, OROV infection is a differential diagnosis in the Amazonas area of Brazil.

 Finally, regard LILACS and IMBIOMED, the number of articles found in them is very limited. In the case of LILACS only 35 and at IMBIOMED none.

 All these comments will be considered in the revised version that will corresponds to version 2 of this manuscript to be uploaded very soon.

# Peer review: Referee response for version 1

**DOI:** 10.5256/f1000research.11788.r20613

**Published:** 2017-03-02

**Authors:** Kateryna Kon

**Affiliations:** 1 Department of Microbiology, Virology and Immunology, Kharkiv National Medical University, Kharkiv, Ukraine

The study is devoted to the bibliometric analysis of research published on the emerging pathogen Oropouche virus. The title of the article is totally appropriate, the abstract provides an adequate summary of the article. There is a comprehensive explanation of study design, methods and analysis used in the study. The conclusions are well balanced and justified on the basis of the results. All sufficient information is provided for replication of results described in the study.

I have read this submission. I believe that I have an appropriate level of expertise to confirm that it is of an acceptable scientific standard.

# Response

**Published:** 2017-03-02

**Authors:** Alfonso Rodriguez-Morales

**Affiliations:** Universidad Tecnológica de Pereira, Colombia

Dear Dr. Kon

 Thank you very much for your comments regard our Research Note on Bibliometrics of Oropouche virus.
